# Time-Lag in Responses of Birds to Atlantic Forest Fragmentation: Restoration Opportunity and Urgency

**DOI:** 10.1371/journal.pone.0147909

**Published:** 2016-01-28

**Authors:** Alexandre Uezu, Jean Paul Metzger

**Affiliations:** 1 Instituto de Pesquisas Ecológicas, Nazaré Paulista, São Paulo, Brazil; 2 Departamento de Ecologia, Instituto de Biociências, Universidade de São Paulo, São Paulo, São Paulo, Brazil; Chinese Academy of Forestry, CHINA

## Abstract

There are few opportunities to evaluate the relative importance of landscape structure and dynamics upon biodiversity, especially in highly fragmented tropical landscapes. Conservation strategies and species risk evaluations often rely exclusively on current aspects of landscape structure, although such limited assumptions are known to be misleading when time-lag responses occur. By relating bird functional-group richness to forest patch size and isolation in ten-year intervals (1956, 1965, 1978, 1984, 1993 and 2003), we revealed that birds with different sensitivity to fragmentation display contrasting responses to landscape dynamics in the Brazilian Atlantic Forest. For non-sensitive groups, there was no time-lag in response: the recent degree of isolation best explains their variation in richness, which likely relates to these species’ flexibility to adapt to changes in landscape structure. However, for sensitive bird groups, the 1978 patch area was the best explanatory variable, providing evidence for a 25-year time-lag in response to habitat reduction. Time-lag was more likely in landscapes that encompass large patches, which can support temporarily the presence of some sensitive species, even when habitat cover is relatively low. These landscapes potentially support the most threatened populations and should be priorities for restoration efforts to avoid further species loss. Although time-lags provide an opportunity to counteract the negative consequences of fragmentation, it also reinforces the urgency of restoration actions. Fragmented landscapes will be depleted of biodiversity if landscape structure is only maintained, and not improved. The urgency of restoration action may be even higher in landscapes where habitat loss and fragmentation history is older and where no large fragment remained to act temporarily as a refuge.

## Introduction

Delays in a species’ response to habitat modification can occur after habitat restoration, when species or communities take longer to reoccupy suitable restored areas (colonization credit) [1–3] or conversely, after habitat loss/fragmentation, when there is a time-lag until species extinctions (extinction debt) [4–6]. The latter occurs when an environmental change restricts resources to a level below those necessary to maintain species viability, although populations persist temporarily in the landscape [4, 6–7]. Such landscapes are important to identify as they must be prioritized in habitat restoration in order to avoid future extinctions.

Although studies during the last two decades revealed general patterns vis-à-vis the time-lag of species responses to habitat alteration, notably relating time-lag with the duration of species life cycle, the degree of habitat disturbance, and the dynamic of spatial structure of habitat patches [4–5, 8–14], we still need to understand several specifics. These include which environmental conditions promote longer lags, which characteristics make species more prone to response delays, and how long these delays last in different species, groups and/or communities [11]. Evaluating the time-lag is difficult because past species composition in a specific location (landscape or habitat patches) is not usually available, forcing researchers to use indirect inferences of time-lag [11]. Additionally, only recently has its importance been realized [12, 15]. Moreover, studies on time-lags in species or community responses to landscape changes are biased to non-tropical regions [16], although it is within the tropics that biodiversity is concentrated and extinction debts (the number of species predicted to go extinct even without further habitat changes [4]) are most likely to occur because of more recent habitat alteration [17].

The time-lag may vary according to the intensity of habitat modification and species characteristics [13, 18–19]. High levels of disturbance generally provoke immediate loss of many species [9, 12, 20], while low or intermediate disturbance leads to longer delays in species responses. A longer relaxation period may occur in long-lived species, such as trees, for example [10, 15]. Species that are just below the critical threshold for metapopulation maintenance may persist longer in fragmented landscapes because of local population dynamics [6–7, 21]. In the latter instance, species idiosyncrasies, such as dispersal capacity, home range, tolerance to edge effects, and demand for specific resource types may drive this dynamic and, consequently, may influence time-lag. Thus, in addition to historic landscape changes, consideration of taxa or species traits is highly relevant [13].

The Atlantic Forest is one of the most threatened tropical regions in the world [22]. Although its original cover has been reduced to 11–16% [23], no cases of bird extinction have been registered so far, a factor potentially explained by time-lags [5], although another possibility is that bird extinctions occurred before scientific documentation of the species. Most of the deforestation in the region occurred from one century to 50 years ago [17, 24–25]. This has led to a high ecological debt, which can be inferred from the high number of threatened species [5] (see also Cowlishaw (1999) for similar examples from Africa [8]). It is therefore essential to consider landscape dynamics in risk evaluations of species in this highly diverse forest [15, 26]. Ignoring the historical changes may be misleading and provoke underestimates of the species threats [7, 10, 27].

We tracked the historical fragmentation of an Atlantic Forest area, quantifying landscape structure in ten-year intervals from the time the area was continuous forest until its contemporary configuration, representing probably the most complete historical landscape mapping of the Atlantic Forest. We sampled bird communities in 28 areas (21 forest fragments and seven areas within a large forest tract). From these data, we evaluated when landscape structure best explained the current richness of bird groups based on ecological species traits, allowing us not only to infer time-lag responses but also to exam whether time-lags vary according to bird traits. We hypothesized that bird groups that differ in sensitivity to habitat modification may display different time-lags in response to forest fragmentation. The most sensitive groups may be more likely to present time-lag when certain conditions of the landscape (e.g., large patches or intermediate degree of isolation) prolong the persistence of these species for some time. These groups are composed of species that present low density, low dispersal capacity, and demand larger areas to survive. On the contrary, the non-sensitive species may accommodate faster to the landscape changes as they generally have higher dispersal capacity and are more flexible to adapt to habitat/landscape changes. We also hypothesized that patch size and isolation may affect time-lag in species responses. This can be the case when the patch is reduced to a size that is smaller than the minimum to maintain viable populations, or when patch isolation is too large to connect functionally the populations/resources that could allow for the persistence in the long term of species in the landscape.

## Methods

### Study area and sites

**Fig 1:**
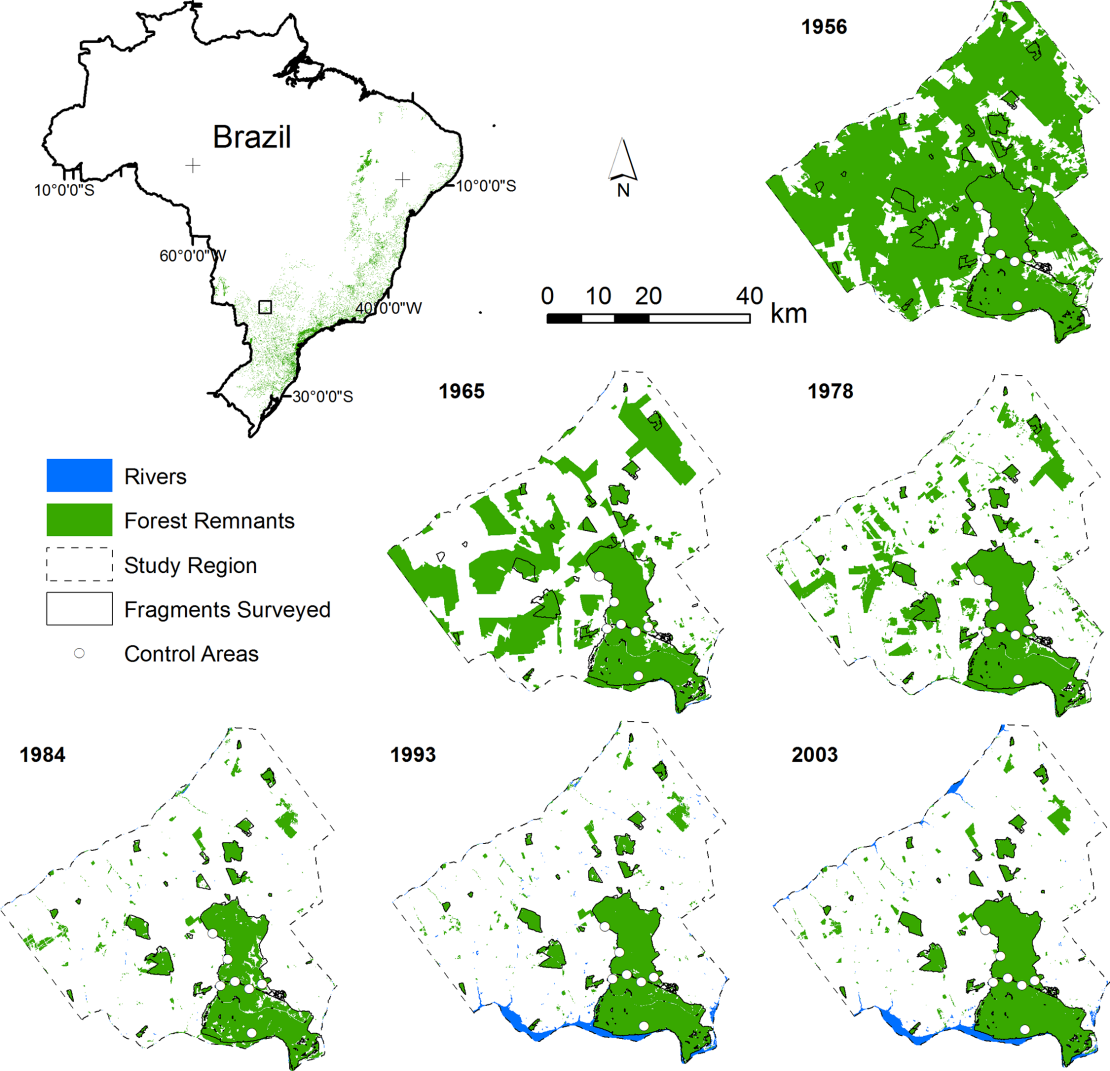
Location of Pontal do Paranapanema region and its changes in forest cover.

The study area, the Pontal do Paranapanema, is situated in the interior of the Atlantic Forest region (52°29’29”W, 22°24’09”S; Fig 1). This area is characterized by a tropical semi-deciduous forest [28], one of the most threatened sub-types within the Atlantic Forest [23]. Deforestation in the area began in the 1950s, mainly for logging and pasture [17]. An important feature of this area is a continuous forest, Morro do Diabo State Park (36,000 ha), which is one of only four patches within this physiognomy larger than 10,000 ha. Adjacent to the park are forest fragments ranging from 2 to 2,000 ha, most of which are within private properties. The remnant forest, including the State Park, now occupies about 18% of the whole region. The matrix is composed mainly of pasture (59.9%) and agriculture (14.6%), both of which are strong impediments to dispersal [29].

Surveys were performed in 28 sites: 21 forest patches and seven sites within the continuous forest of Morro do Diabo State Park (Fig 1). To ensure a range of fragment sizes to represent the entire set of relevant patches for bird conservation in the region, we selected seven large (400–1500 ha), seven medium (100–200 ha) and seven small fragments (30–80 ha). We did not chose patches smaller than 30 ha because generally these patches are much degraded due to the edge effect, which is very intense in this type of tropical semi-deciduous forest. We verified such a condition in the region based on imagery analysis and field visits.

Pontal do Paranapanema is an excellent region to study the species’ time lag response to habitat fragmentation, as there are good records of landscape modification (in aerial photos and satellite images) since it was a ~200,000 ha block of continuous forest. Furthermore, during its history the landscape matrix was maintained as homogenous, composed of pasture and sugarcane with low human density, which permits us to keep the analysis relatively simple as there are few other factors influencing species richness in the region besides the spatial structure of forest patches and its dynamics.

### Biological survey and classification

Bird surveys were conducted using the point count methodology [30] during three reproductive seasons from 2003 to 2005, with each site visited eight times. Sampling was conducted during the first three morning hours after sunrise. At each sample site, six points were selected 200 m apart in a rectangular 3 x 2 format quadrat. During each visit, every point was observed during ten minutes. Only one person (A.U.) did the survey in order to avoid bias provoked by different bird identification capacities. Bird songs not immediately recognized in the field were recorded for later identification. We did not include non-forest-dependent species (according to [31] and field observations) and those that did not adhere to the sample criteria, species that are nocturnal or vocalize inconspicuously. The permit for bird surveys in the Morro do Diabo State Park was provided by Instituto Florestal (Forest Institute, COTEC n. 42.125/2002), from the Environmental Department of the State of São Paulo, and in private land, permissions were given by all landowners. As this study was observational and did not involve collecting birds, all the necessary permissions were related exclusively to the access of the researcher in the areas and this study did not involve endangered species.

To verify which species traits might be associated with time-lags in bird responses, we categorized taxa into groups defined according to six criteria. *Proximity to the edge of distribution* was determined using species-distribution maps [32] by measuring the distance (d) from a central point in the study region to the nearest limit of a species distribution. We separated species into two categories: near the edge (d < 200 km) and far from the distribution edge (d > 200 km). *Degree of endemism* was defined by a taxon’s status as endemic or non-endemic to the Atlantic Forest [31]. *Center of abundance* in lowland (<500 m) or highland (> 500 m) was adapted from [31]. *Flexibility in the use of different forest types* was subdivided into species that occur in 1–2 types, 3–4 types, and 5–6 types of forest [31]. The *relative abundance* criterion was defined as low, medium or high [31]. *Guilds* were: 1. carnivores, 2. large canopy frugivores and omnivores, 3. large canopy insectivores, 4. edge insectivores, 5. understory insectivores, 6. canopy insectivores, 7. edge frugivores and omnivores, 8. understory omnivores and 9. large ground insectivores and frugivores [31, 33]. Uezu and Metzger (2011) observed previously that these traits are related to bird sensitivity to habitat reduction and/or fragmentation, and the following groups were the most affected by landscape changes: Atlantic Forest endemics, species proximal to their distribution limit, those with low forest-type flexibility (1–2 types), highlands species, understory insectivores, understory omnivores, and large terrestrial [29]. For our analysis, we considered both strictly forest-dependent and generalist species. We considered generalist those forest species that are also able to use open areas.

### Landscape structural analyses

The study area was mapped for six different years: 1956, 1965, 1978, 1984, 1993 and 2003. We interpreted aerial photographs to map the study area in 1956 using ArcGIS (scale 1:35,000). Data from 1965 and 1978 were collected by digitalization of topographic maps (scales 1:50,000 and 1:10,000; respectively). We classified LANDSAT 5 satellite images for 1984, 1993 and 2003. For each year, we mapped two categories of occupation: forest and non-forest. Maps were geo-referenced using the same projection and datum and, because the sources were of different resolution, we converted these to raster type and resampled them for a pixel size of 30 m (which corresponded to the lowest map resolution) prior to the analysis. In order to remove small patches, we used a low-pass filter (3 x 3 pixel) and, additionally, eliminated patches smaller than 1 ha. Since the beginning of substantial human occupation in the early 50’s, there has been a great contrast between the natural forest and the surrounding matrix (generally composed of pasture and sugar cane), which facilitates the delimitation of land use and land cover types and reduced the chances of mapping errors.

To quantify the deforestation process, we calculated landscape indexes at regional and local scales. At a regional level, we considered an area of about 250,000 ha, which correspond to the mapped area of 1956 (Fig 1). The metrics were: 1. *number of patches* (NP); 2. *mean largest patches size* (MLP), mean of the tenth largest patches, excluding the control site; 3. *percentage of area occupied by forest* (PF%); and 4. *degree of isolation*, which is the mean Euclidean nearest neighbor distance from all forest fragments in the landscape (ENN_MN; m). For the latter metric, we eliminated all fragments smaller than 10 ha to suppress the effect of small patches. These indices give a general description of the landscape structure and allow a quantitative comparison of the landscape along time.

At a local level, we considered metrics that are usually associated with the occurrence of bird species in fragmented landscapes: patch size and isolation. Those metrics may have different effects in different bird groups. Patch area might be more relevant for species with a restricted dispersal capacity, particularly those that rarely cross open areas [29]. For species that can overpass partially the matrix, the degree of isolation might be more relevant, affecting the capacity of the species to use a cluster of patches to achieve their food demands. We selected two indexes that present low correlation along the studied period (R Pearson = 0.24–0.35): logarithm of the area (AREA) and the logarithm of proximity (PROX). The latter consists of the sum of all forest fragment areas within a radius of 2 km from the edge of the focal fragment, divided by the squared distance, edge-to-edge, between these fragments [34].

### Data Analysis

Bird richness (number of species, i.e. alpha diversity) was obtained from the survey data of species groups. To find evidence for time lag in species responses, we related bird richness indices with landscape variables (AREA and PROX, log transformed) from present and past landscapes. If past landscape parameters best explain the present-day pattern of bird richness, we considered that bird groups are still responding to the past landscape and time lag exists (a similar approach was used in other studies [10, 14]). It is expected that both landscape variables (size and isolation) can affect time lag, as expressed in the hypotheses. The interaction between these variables is also important, because they both can regulate key processes that affects population and metapopulation survival.

We only considered landscape indices from 1965, 1978 and 2003 because landscape structure remained relatively stable from 1984 to 2003, and landscape indices were highly correlated among each other (r > 0.98 for patch area and r > 0.99 for degree of proximity). Moreover, in 1956 most studied fragments belonged to the same continuum, preventing any statistical analysis. We proposed 13 Generalized Linear Models: a null model; six models for each temporal studied period (1965, 1978 and 2003) considering one explicative variable at a time (AREA or PROX); and six models considering both landscape metrics from each corresponding year, allowing for additive and interactive models (Table 1). Error structure was assumed to have a Poisson distribution.

**Table 1:** Landscape variables for the nine candidate models of linear regression: multiple (m01–m03, considering only the additive effect of explicative variables and m10 –m12, considering the interactive effect of explicative variables) and simple regression (m04–m09). The variables AREA and PROX represent the logarithm of patch area and degree of proximity calculated for different dates in the Pontal do Paranapanema, Brazil.

Models	Var. 1	Var. 2	Type
**m00**			Null model
**m01**	AREA65	PROX65	Additive effect
**m02**	AREA78	PROX78	Additive effect
**m03**	AREA03	PROX03	Additive effect
**m04**	AREA65		Single variable
**m05**	AREA78		Single variable
**m06**	AREA03		Single variable
**m07**		PROX65	Single variable
**m08**		PROX78	Single variable
**m09**		PROX03	Single variable
**m10**	AREA65	PROX65	Interactive effect
**m11**	AREA78	PROX78	Interactive effect
**m12**	AREA03	PROX03	Interactive effect

To identify the model that best fits, we used AICc–Akaike’s Information Criteria, adjusted for small sample sizes [35]. This procedure ranks the models indicating those with higher probabilities to be selected according to the sample data set. In this analysis, we calculated: AICc–estimation of the relative distance between a candidate model and the “real” model; Δi AIC–relative difference of AICc value (from a certain model) in relation to the smaller value of AICc among all models and w AICc–chance for the model to be selected, which varies from 0 to 1. The pseudo R-squared (pseudo coefficient of determination [36]) and the relative importance of the variables were also calculated. The latter corresponds to the sum of w AICc of all models in which a variable was included [35].

Finally, to indicate the patches with a higher potential to hold more species that have yet to stabilize but may be lost in the near future in the region, we used the regression model that best explained the variation of sensitive bird richness to estimate the number of sensitive species in all forest patches in the region. This analysis illustrated how we can use this type of information of landscape dynamics to set priority areas for conservation and restoration.

Bird and landscape data for open-access use are available online at the Dryad Digital Repository: doi:10.5061/dryad.f180j

## Results

### The fragmentation process

**Fig 2:**
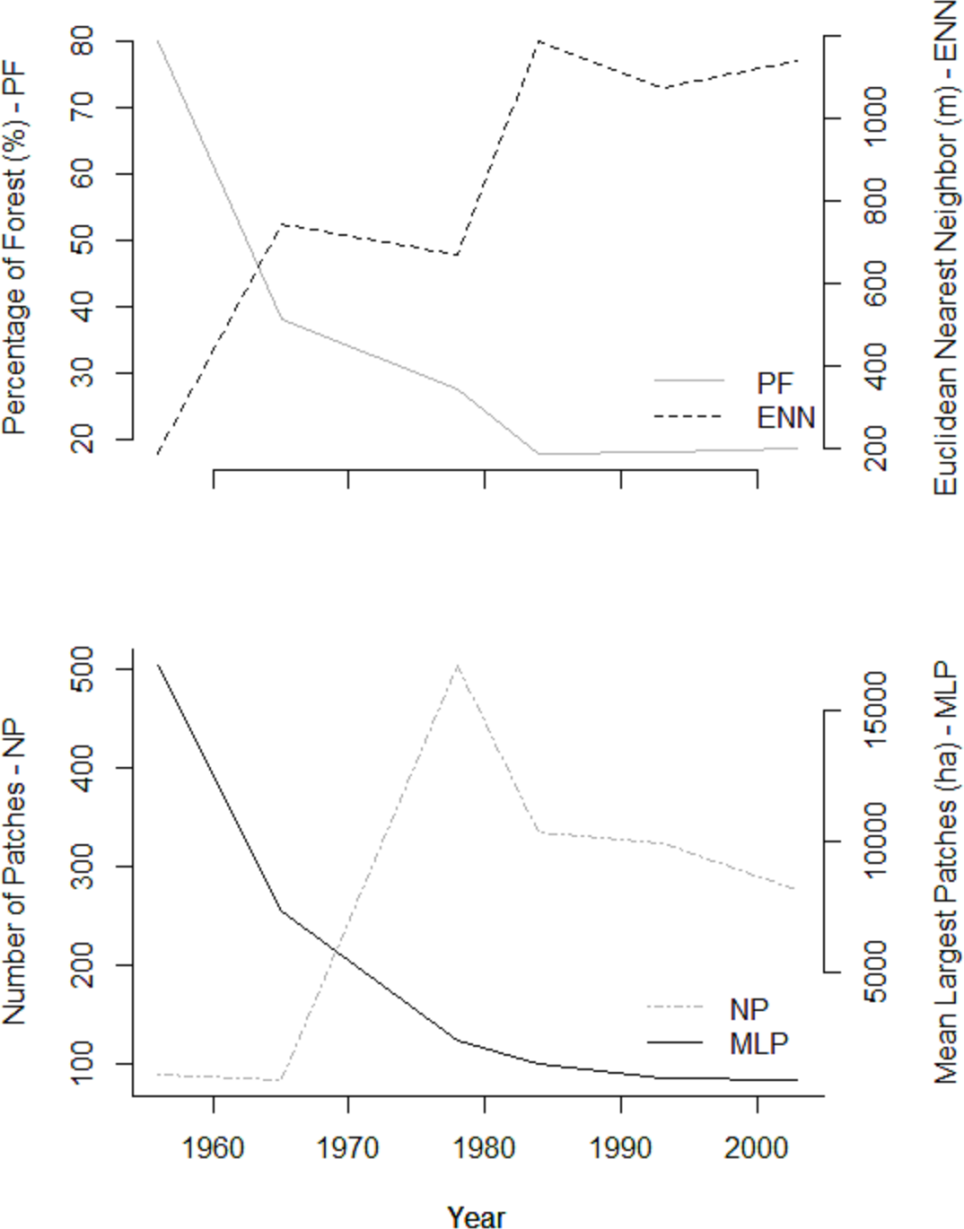
Variation across time in percentage of forest (PF), the mean Euclidean distance to nearest neighbor among the patches (ENN), number of patches (NP), and mean largest patches size (MLP), in Pontal do Paranapanema region.

In 1956, the Pontal do Paranapanema was approximately 80% covered with forest, mostly arranged in a continuous expanse of more than 200,000 ha (Fig 1). All the patches considered in this study were connected to each other in 1956. The landscape underwent considerable change beginning in 1965, with the proportion of forest cover reduced to approximately 40%. This resulted in a fragmented landscape with many isolated patches, but these comprised large forests. However, the region still harbored twice as much forest cover as compared to today. In 1965, some patches analyzed herein were still interconnected, but many had already become isolated (Figs 1 and 2).

In 1978, the percentage of forest was 27.5%. Many of the previously large remnants had been replaced by many smaller patches. During this period, the region underwent the highest level of fragmentation. There were more patches in the landscape, and mean patch size was substantially reduced, although the current largest patches were still larger then (Fig 2). The decade around 1978 saw deforestation shape the landscape into the currently recognizable forest patches (Fig 1). It was also during this period when patch isolation reached a plateau. In 1984, the region comprised less than 18% forest, the lowest level recorded in this study. Since then, there has been a slight increase in forest coverage, reaching 18.2% in 1993 and, almost 19% in 2003 (Fig 2).

### Structure of the bird community

We registered 159 bird species, but considered only 99 strictly forest-dependent and 19 generalists ones. The group richness based on ecological traits varied considerably among patches and bird communities showed no autocorrelation among study sites, as verified in previous work [29] (S1 Appendix).

### Most important models and parameters

Models for 1978 had the highest chance of being selected and better explained the variation of many bird groups’ richness (Table 2): Atlantic Forest endemics, species proximal to their distribution limit, those with low forest-type flexibility (1–2 types), highlands species, understory insectivores and understory omnivores. These groups encompass all species traits that were related with high sensitivity to habitat fragmentation in our previous study [29], suggesting that sensitive groups’ richness is more closely related to the landscape of ~25 years before the bird survey was conducted than to its present-day configuration. For those groups, patch area was a more relevant explicative variable than proximity. The large ground insectivores and frugivores was the only sensitive group that was not related to 1978 landscape parameters. In addition, the interaction between the independent variables (patch area and proximity) was not relevant to explain bird richness.

**Table 2:** Best regression models selected (Δi AICc < 2) that explain the variation of *bird groups’ richness*, using AICc, including: pseudo R^2^ –coefficients of determination; AICc–the model distance to the “real” model; Δi AICc–relative value of AICc and w AICc–Akaike’s weight, chance for the model to be selected.

Bird groups	Independent variables	Pseudo R^2^	AICc	Δi AIC	w AIC
Endemic	AREA78+PROX78	0.80	85.5	0.00	0.41
Endemic	AREA78	0.72	85.7	0.17	0.38
1–2 types of forest	AREA78	0.58	115.0	0.00	0.37
1–2 types of forest	AREA78+PROX78	0.65	116.0	1.13	0.21
Low abundance	AREA78	0.44	78.6	0.00	0.41
Low abundance	AREA78+PROX78	0.54	79.3	0.73	0.29
High land	AREA78	0.55	114.0	0.00	0.45
High land	AREA78+PROX78	0.60	114.0	0.51	0.35
Edge (<200 km)	AREA78+PROX78	0.60	116.0	0.00	0.47
Edge (<200 km)	AREA78	0.48	117.0	1.25	0.25
Understory insectivores	AREA78	0.55	109.0	0.00	0.41
Understory insectivores	AREA78+PROX78	0.62	110.0	0.28	0.35
Understory omnivores	AREA78+PROX78	0.76	26.7	0.00	0.51
Understory omnivores	AREA78*PROX78	0.81	28.5	1.76	0.21
Large ground insetivores frugivores	NULL	0.00	53.9	0.00	0.29
Non endemic	PROX03	0.22	154.0	0.00	0.50
≥3 types of forest	PROX03	0.16	146.0	0.00	0.34
≥3 types of forest	NULL	0.00	148.0	1.68	0.15
High abundance	AREA78	0.25	131.0	0.00	0.25
High abundance	PROX03	0.21	131.0	0.72	0.17
High abundance	AREA03	0.20	132.0	1.08	0.14
High abundance	AREA03+PROX03	0.32	132.0	1.47	0.12
Medium abundance	PROX03	0.26	126.0	0.00	0.26
Medium abundance	NULL	0.00	128.0	1.40	0.13
Low land	PROX03	0.23	149.0	0.00	0.57
Center (>200 km)	PROX03	0.19	146.0	0.00	0.35
Center (>200 km)	NULL	0.00	147.0	1.95	0.13
Carnivores	NULL	0.00	61.7	0.00	0.30
Carnivores	AREA03	0.13	63.3	1.54	0.14
Carnivores	AREA78	0.08	63.7	1.98	0.11
Large canopy frugivores omnivores	AREA78	0.37	105.0	0.00	0.19
Large canopy frugivores omnivores	AREA65	0.33	106.0	0.32	0.17
Large canopy frugivores omnivores	NULL	0.00	106.0	0.46	0.15
Large canopy insectivores	NULL	0.00	79.7	0.00	0.31
Edge insectivores	AREA78	0.37	94.1	0.00	0.24
Edge insectivores	AREA03	0.32	94.5	0.45	0.19
Edge insectivores	NULL	0.00	94.7	0.64	0.18
Canopy insectivores	PROX03	0.30	95.3	0.00	0.44
Edge frugivores omnivores	NULL	0.00	112.0	0.00	0.30

In contrast, for non-sensitive bird groups the responses were more heterogeneous, *i*.*e*. there was a higher uncertainty in model selection and for many groups the null model was among those selected. In general, the w AIC was low and not very different among candidate models. The pseudo coefficients of determination (pseudo R-square) are also low (Table 2), indicating that landscape parameters poorly explain the variation in richness of these bird groups. Nevertheless, for some non-sensitive groups, models including the more recent (2003) proximity variable had the highest chance to be selected.

The relative importance of the variables confirms that the most sensitive bird groups respond most to 1978 patch sizes, whereas for some non-sensitive groups, the 2003 proximity variable was of relatively high importance (Table 3).

**Table 3:** Relative Importance of explicative variable from nine candidate models: sum of w AICc (chance of a model to be selected, which range from 0 to 1) of all the models where a variable were included. Higher values define more important variables. Values higher than 0.50 are in boldface.

Bird groups	area65	prox65	area78	prox78	area03	prox03
Endemic	0.00	0.00	**0.86**	0.49	0.14	0.05
1–2 types of forest	0.06	0.02	**0.63**	0.26	0.29	0.18
Low abundance	0.01	0.00	**0.79**	0.41	0.15	0.07
High land	0.06	0.03	**0.89**	0.44	0.05	0.03
Edge (<200 km)	0.01	0.00	**0.82**	**0.58**	0.16	0.10
Understory insectivores	0.01	0.00	**0.82**	0.42	0.17	0.09
Understory omnivores	0.00	0.00	**0.78**	**0.72**	0.22	0.16
Large ground insectivores and frugivores	0.14	0.11	0.17	0.11	0.16	0.11
Non endemic	0.06	0.04	0.10	0.07	0.22	**0.68**
≥3 types of forest	0.08	0.07	0.14	0.11	0.18	0.45
High abundance	0.10	0.05	0.31	0.10	0.28	0.31
Medium abundance	0.12	0.10	0.14	0.15	0.18	0.36
Low land	0.03	0.03	0.05	0.05	0.20	**0.73**
Center (>200 km)	0.10	0.06	0.13	0.08	0.19	0.47
Carnivores	0.13	0.12	0.14	0.12	0.17	0.11
Large canopy frugivores and omnivores	0.21	0.09	0.26	0.17	0.16	0.11
Large canopy insectivores	0.10	0.11	0.12	0.20	0.12	0.15
Edge insectivores	0.06	0.06	0.32	0.17	0.25	0.10
Canopy insectivores	0.05	0.14	0.09	0.14	0.15	**0.56**
Edge frugivores and omnivores	0.11	0.13	0.13	0.20	0.11	0.12

## Discussion

### Distinctive time-lags between sensitive and non-sensitive bird groups

Species groups displayed different responses to the fragmentation dynamics. Non-sensitive species generally presented a wider distribution and higher frequency in the sampled areas, reflecting a higher capacity to adapt to novel habitat arrangements. This flexibility would explain the lower correlation between the richness of these groups and the landscape parameters. Moreover, many of these species are less averse to crossing open areas than the more sensitive species [37–38], increasing their rates of dispersal between patches and, consequently, making *proximity* a more important parameter for explaining distribution. In another Atlantic Forest landscape [15], species with higher dispersal capacity had stronger responses to habitat connectivity. Moreover, higher dispersal rates would make them less likely to experience a time-lag, as suggested by Lindborg and Eriksson (2004) [10].

Although the recent landscape partly explains the species richness of sensitive birds in forest fragments [29], we observed that landscape parameters from 1978, especially patch size, better fit their spatial pattern of richness. These groups are composed of species with lower dispersion capacity (understory insectivores, understory omnivores, and large terrestrial), with naturally low abundance (Atlantic Forest endemics, species proximal to their distribution limit, and highlands species) and with higher demands for habitat (those with low forest-type flexibility, 1–2 types). Our results indicate that the diversity of these groups did not stabilize, and more losses are likely as the communities relax (even without further landscape changes), indicating a higher risk for these taxa. The data about past landscape structure contributed to our understanding of the current process of species losses [6, 10], suggesting that landscape management is imperative to avoid future extinctions.

When the species are ranked according to their sensitivities (as observed in the study region and described in the literature), we can speculate which species are more susceptible to local extinction following community relaxation in forest fragments. There are two groups especially threatened: those that we verified are very affected by forest fragmentation and those previously confirmed in the region in other studies but that were not observed during the surveys, potentially because of low density. These species have many characteristics that correlate to a higher sensitivity, such as endemism to the Atlantic Forest and low natural abundance (Table 4). Many of these taxa are already locally extinct in other Atlantic Forest regions [39–41], highlighting the real possibility of similar losses in the Pontal do Paranapanema region.

**Table 4:** List of species probably most susceptible to local extinction following community relaxation in forest fragments.

Family	English	Species	Status in the study	Endemism	Natural Abundance
Tinamidae	Solitary Tinamou	*Tinamus solitarius*	Low Abundance	Yes	Low
Odontophoridae	Spot-winged Wood-Quail	*Odontophorus capueira*	Low Abundance	Yes	Medium
Cotingidae	Shrike-like Cotinga	*Laniisoma elegans*	Low Abundance	No	Medium
Cotingidae	Wing-barred Piprites	*Piprites chloris*	Low Abundance	No	Medium
Cotingidae	Bare-throated Bellbird	*Procnias nudicollis*	Low Abundance	Yes	Medium
Falconidae	Barred forest-falcon	*Micrastur ruficollis*	Low Abundance	No	Medium
Formicariidae	Eye-ringed Tody-Tyrant	*Hemitriccus orbitatus*	Low Abundance	Yes	Medium
Formicariidae	Short-tailed antthrush	*Chamaeza campanisona*	Low Abundance	No	Medium
Furnariidae	Rufous-breasted leaftosser	*Sclerurus scansor*	Low Abundance	Yes	Low
Parulidae	White-browed Warbler	*Myiothlypis leucoblephara*	Low Abundance	Yes	High
Psittacidae	Red-and-green Macaw	*Ara chloropterus*	Low Abundance	No	Medium
*Accipitridae*	Ornate Hawk-Eagle	*Spizaetus ornatus*	Not detected	No	Low
*Accipitridae*	Black-and-white hawk eagle	*Spizaetus melanoleucus*	Not detected	No	Low
*Cotingidae*	Red-ruffed Fruitcrow	*Pyroderus scutatus *	Not detected	Yes	Low
*Ramphastidae*	Black-necked Aracari	*Pteroglossus aracari*	Not detected	No	High
*Ramphastidae*	Saffron Toucanet	*Pteroglossus bailloni*	Not detected	Yes	Medium
*Ramphastidae*	Spot-billed Toucanet	*Selenidera maculirostris*	Not detected	Yes	Low
*Ramphastidae*	Red-breasted Toucan	*Ramphastos dicolorus*	Not detected	Yes	Medium

### Fragmentation threshold and the time-lag

Based on the inferred history of the Pontal do Paranapanema region and on the estimated 25-year time-lag, we posit that sensitive groups are still responding to events that occurred between 1965 and 1978, when the landscape became highly modified. It follows that many species should go extinct in a near future if landscape structure is not improved by restoration action [42]. Although landscapes with 10 to 30% habitat cover experience rapid biodiversity decline [18, 43–48], many species persist even after habitat reduction and isolation, resulting in an extinction debt.

In the Pontal do Paranapanema, two conditions were essential to create extinction debts: the maintenance of large patches in the landscapes and the high degree of isolation of bird communities. The mean largest patch sizes in the Pontal do Paranapanema declined by almost three times between 1978 and 2003. However, despite this reduction, there are still large patches (~ 100 ha) in the fragmented landscape allowing to maintain the populations of those sensitive specie just below their minimum requirements. Larger patches (including the Park used as a control) allow for longer persistence of local populations, where sensitive species become concentrated in low density. As a consequence, time-lag is longer in these conditions [6, 11]. Conversely, species loss is more rapid in smaller patches, which we observed in the fewer number of sensitive species in small and medium patches. We did not survey patches smaller than 30 ha, but probably they have experienced a much faster species loss than in the patches we have included in the analysis.

Moreover, concerning the isolation of bird communities, in 1978 the mean distance between forest fragments was already far from each other, approximately 700 m, longer than the distance that most forest bird species will travel (e.g., < 100 m; [49–52]. This high degree of isolation makes the species more dependent on the forest patches they already occupied, which can explain why sensitive groups were more affected by fragment size. If patch isolation were less intense, higher rates of dispersal would increase persistence in fragments, resulting in longer time-lags [6, 11] or even in stable viable populations. In this last situation, sensitive species would show the same pattern as non-sensitive groups, being affected by current proximity and not presenting evidence of time-lag.

### Restoration opportunity and urgency

The history of human settlement in the Pontal do Paranapanema during the last fifty years reflects that of the entire Atlantic Forest. In both cases, forest cover is below a critical 30% extinction threshold, observed previously for birds in the Atlantic forest [53]. Despite this high level of deforestation, no bird extinctions were detected in the Atlantic Forest [5]. The main evidence of an extinction debt is the number of threatened species, consistent with what is predicted to go extinct by species-area relationships [5]. These taxa are primarily concentrated in large continuous forests, or in particular habitats that support their long-term presence [50, 54]. However, in the many regions without such remnants, these species have already gone locally extinct [39–40]. These results emphasize the benefits of keeping large patches in regional or landscape scales. Even in highly-degraded regions with low overall forest cover (<30%), these patches can support (for some time) the presence of many sensitive species even after years of isolation, being a refuge for many species that can be rescued by future restoration actions.

**Fig 3:**
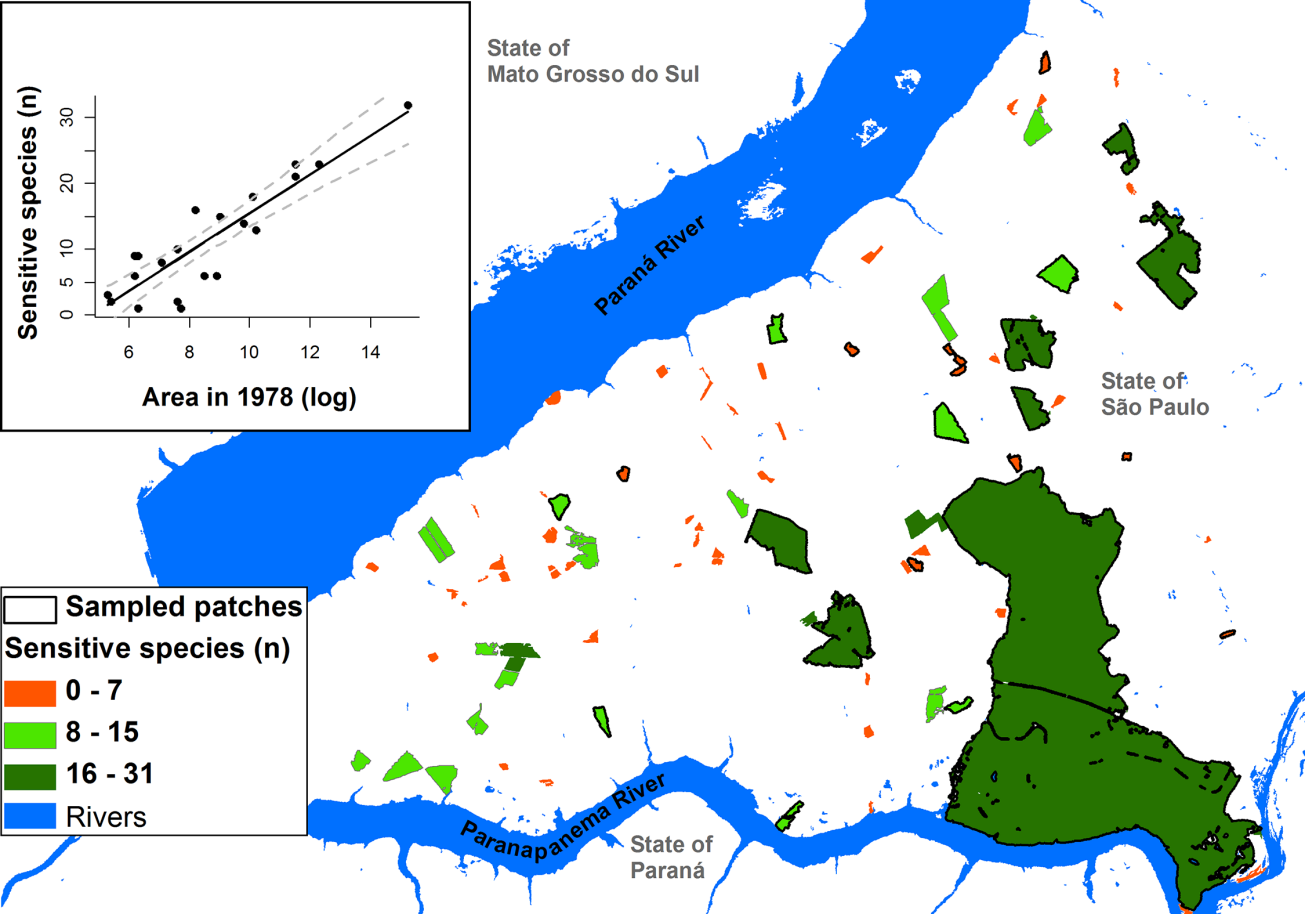
Potential extinction debt estimated by the number of sensitive bird species, based on 1978 patch size as predictive variable (model in the chart) in Pontal do Paranapanema, SP-Brazil.

Our results emphasize the need to consider historical landscape changes to inform how fragmentation threatens species. It is also a way to predict where future extinctions will occur, so we can anticipate and prioritize the actions needed to avoid them [55]. For instance, we used the 28 studied sites to infer extinction debts for all patches in Pontal do Paranapanema (Fig 3). With this information, we suggest two restoration actions to neutralize these debts: to expand the largest fragments, aiming to reach a size comparable to the fragments existing in 1978, or alternatively, to connect these forest patches (e.g. with the creation of corridors), reducing the dependence of species on single forest patches and increasing the chance of forest colonization. In both cases, the plan would be to recreate a landscape’s condition for viable populations, and to avoid paying the extinction debt.

In most parts of the Atlantic Forest, the situation may be more critical. This could be particularly the case in areas where the critical value of fragmentation was reached more than 50 years ago and where no larger fragment is left. Time-lags likely also vary across the Atlantic Forest because of the range of landscapes, from more connected [15, 40, 47, 50] to more isolated [39, 56]. In landscapes with small and highly isolated patches, fragmentation effects should be immediate [11]. Nonetheless, we expect longer responses in landscapes where conditions are beyond the threshold to maintain viable (meta) populations [6–7]. In our study, we found strong evidence that large patch size can create such a condition. These landscapes must hold the most threatened populations and, therefore, are where (or from where) we should concentrate restoration actions.

## Conclusion

By relating the richness of sensitive species to present and past landscape structure, it is possible to consider past landscape dynamics in species risk evaluation and extinction debt estimation, identifying priority areas for restoration actions. Our data showed that birds sensitive to fragmentation effects present a 25 years time-lag to landscape changes, reinforcing the idea that it is not enough to conserve the present landscape structure to maintain those species in the long term. It is necessary and urgent to improve this structure through landscape restoration, enlarging or improving the best and largest fragments, and increasing connectivity among those fragments [57–59]. This approach can be useful to optimize conservation funds and rescue species from extinction in highly endangered and biodiverse systems, such as the Atlantic Forest [55].

## Supporting Information

S1 Appendix List of studied forest species with their sensibility to habitat loss and fragmentation. (DOC)
