# Supplementary material for: Time-Lag in Responses of Birds to Atlantic Forest Fragmentation: Restoration Opportunity and Urgency
Source: PLoS One. 2016 Jan 28;11(1):e0147909. doi: 10.1371/journal.pone.0147909 (PMC4731062; doi:10.1371/journal.pone.0147909)
Supplement: S1 Appendix — (DOC) [file pone.0147909.s001.doc]

**S1 Appendix** – List of studied forest species with their sensibility to habitat loss and fragmentation. Also listed are the categories of functional groups into which we classified them for the Pontal do Paranapanema region, Southeastern, Brazil.

| **Species** | **Endemism** | **Limit of Distribution** | **Type of forest** | **Relative abundance** | **Center of abundance** | **Guilds** |
| --- | --- | --- | --- | --- | --- | --- |
| *Procnias nudicollis* | Endemic | < 200 km | 1 - 2 | Medium | High Lands | Large Canopy Frug. and Omn. |
| *Automolus leucophthalmus* | Endemic | < 200 km | 1 - 2 | Medium | Low Lands | Understory insectivores |
| *Hemitriccus orbitatus* | Endemic | < 200 km | 1 - 2 | Medium | Low Lands | Understory insectivores |
| *Xiphorhynchus fuscus* | Endemic | < 200 km | 1 - 2 | Medium | Low Lands | Understory insectivores |
| *Malacoptila striata* | Endemic | < 200 km | 1 - 2 | Low | Low Lands | Understory insectivores |
| *Pyriglena leucoptera* | Endemic | < 200 km | 1 - 2 | High | Low Lands | Understory insectivores |
| *Sclerurus scansor* | Endemic | < 200 km | 1 - 2 | Low | Low Lands | Understory insectivores |
| *Tinamus solitarius* | Endemic | < 200 km | 1 - 2 | Low | Low Lands | Large ground insect. and frug. |
| *Pyrrhura frontalis* | Endemic | < 200 km | 3 - 4 | High | High Lands | Large Canopy Frug. and Omn. |
| *Basileuterus leucoblepharus* | Endemic | < 200 km | 3 - 4 | High | High Lands | Understory insectivores |
| *Chiroxiphia caudata* | Endemic | < 200 km | 3 - 4 | High | High Lands | Understory omnivores |
| *Baryphthengus ruficapillus* | Endemic | < 200 km | 3 - 4 | High | Low Lands | Understory insectivores |
| *Trogon surrucura* | Endemic | > 200 km | 1 - 2 | High | High Lands | Large Canopy Frug. and Omn. |
| *Odontophorus capueira* | Endemic | > 200 km | 1 - 2 | Medium | High Lands | Large ground insect. and frug. |
| *Philydor lichtensteini* | Endemic | > 200 km | 1 - 2 | High | Low Lands | Understory insectivores |
| *Schiffornis virescens* | Endemic | > 200 km | 3 - 4 | Medium | High Lands | Understory omnivores |
| *Laniisoma elegans* | Non-endemic | < 200 km | 1 - 2 | Low | High Lands | Understory omnivores |
| *Chamaeza campanisona* | Non-endemic | < 200 km | 1 - 2 | Medium | High Lands | Large ground insect. and frug. |
| *Crypturellus obsoletus* | Non-endemic | < 200 km | 1 - 2 | Medium | High Lands | Large ground insect. and frug. |
| *Trogon rufus* | Non-endemic | < 200 km | 1 - 2 | Low | Low Lands | Large Canopy Frug. and Omn. |

**S1 Appendix** – continuation

| **Species** | **Endemism** | **Limit of Distribution** | **Type of forest** | **Relative abundance** | **Center of abundance** | **Guilds** |
| --- | --- | --- | --- | --- | --- | --- |
| *Herpsilochmus atricapillus* | Non-endemic | < 200 km | 1 - 2 | High | Low Lands | Canopy insectivores |
| *Piprites chloris* | Non-endemic | < 200 km | 1 - 2 | Medium | Low Lands | Canopy insectivores |
| *Habia rubica* | Non-endemic | < 200 km | 1 - 2 | Medium | Low Lands | Understory omnivores |
| *Turdus albicollis* | Non-endemic | < 200 km | 1 - 2 | Medium | Low Lands | Understory omnivores |
| *Lurocalis semitorquatus* | Non-endemic | < 200 km | 1 - 2 | Low | Low Lands | NA |
| *Amazona amazonica* | Non-endemic | < 200 km | 3 - 4 | Medium | Low Lands | Large Canopy Frug. and Omn. |
| *Celeus flavescens* | Non-endemic | < 200 km | 3 - 4 | Medium | Low Lands | Large Canopy insectivores |
| *Cissopis leverianus* | Non-endemic | < 200 km | 3 - 4 | Medium | Low Lands | Understory insectivores |
| *Manacus manacus* | Non-endemic | < 200 km | 3 - 4 | Medium | Low Lands | Understory omnivores |
| *Herpsilochmus rufimarginatus* | Non-endemic | < 200 km | 5 - 6 | High | High Lands | Canopy insectivores |
| *Dysithamnus mentalis* | Non-endemic | > 200 km | 1 - 2 | High | High Lands | Understory insectivores |
| *Platyrinchus mystaceus* | Non-endemic | > 200 km | 1 - 2 | Medium | High Lands | Understory insectivores |
| *Micrastur ruficollis* | Non-endemic | > 200 km | 1 - 2 | Medium | Low Lands | Carnivores |
| *Notharcus swainsoni* | Non-endemic | > 200 km | 1 - 2 | Medium | Low Lands | Large Canopy insectivores |
| *Corythopis delalandi* | Non-endemic | > 200 km | 1 - 2 | High | Low Lands | Understory insectivores |
| *Leptopogon amaurocephalus* | Non-endemic | > 200 km | 1 - 2 | Medium | Low Lands | Understory insectivores |
| *Sirystes sibilator* | Non-endemic | > 200 km | 1 - 2 | Low | Low Lands | Canopy insectivores |
| *Pionus maximiliani* | Non-endemic | > 200 km | 3 - 4 | Medium | High Lands | Large Canopy Frug. and Omn. |
| *Basileuterus culicivorus* | Non-endemic | > 200 km | 3 - 4 | High | High Lands | Understory insectivores |
| *Xiphocolaptes albicollis* | Non-endemic | > 200 km | 3 - 4 | Low | High Lands | Understory insectivores |
| *Xenops rutilans* | Non-endemic | > 200 km | 3 - 4 | Medium | High Lands | Canopy insectivores |
| *Dendrocolaptes platyrostris* | Non-endemic | > 200 km | 3 - 4 | Medium | Low Lands | Understory inseticvores |

**S1 Appendix – continuation**

| **Species** | **Endemism** | **Limit of Distribution** | **Type of forest** | **Relative abundance** | **Center of abundance** | **Guilds** |
| --- | --- | --- | --- | --- | --- | --- |
| *Cacicus haemorrhous* | Non-endemic | > 200 km | 3 - 4 | Medium | Low Lands | Edge frugivores and omnivores |
| *Claravis pretiosa* | Non-endemic | > 200 km | 3 - 4 | Medium | Low Lands | Edge frugivores and omnivores |
| *Leptotila rufaxilla* | Non-endemic | > 200 km | 3 - 4 | High | Low Lands | Edge frugivores and omnivores |
| *Crypturellus tataupa* | Non-endemic | > 200 km | 3 - 4 | Medium | Low Lands | Large ground insect. and frug. |
| *Parula pitiayumi* | Non-endemic | > 200 km | 5 - 6 | High | High Lands | Canopy insectivores |
| *Sittasomus griseicapillus* | Non-endemic | > 200 km | 5 - 6 | High | Low Lands | Understory insectivores |
| *Vireo chivi* | Non-endemic | > 200 km | 5 - 6 | High | Low Lands | Edge frugivores and omnivores |
| *Synallaxis frontalis* | Non-endemic | < 200 km | 3 - 4 | Medium | Low Lands | Edge insectivores |
| *Taraba major* | Non-endemic | < 200 km | 3 - 4 | High | Low Lands | Understory insectivores |
| *Myiarchus tyrannulus* | Non-endemic | < 200 km | 5 - 6 | Medium | Low Lands | Edge insectivores |
| *Amazona aestiva* | Non-endemic | > 200 km | 1 - 2 | Medium | Low Lands | Large Canopy Frug. and Omn. |
| *Patagioenas picazuro* | Non-endemic | > 200 km | 1 - 2 | High | Low Lands | Large Canopy Frug. and Omn. |
| *Myiopagis caniceps* | Non-endemic | > 200 km | 1 - 2 | Medium | Low Lands | Canopy insectivores |
| *Pitangus sulphuratus* | Non-endemic | > 200 km | 1 - 2 | High | Low Lands | Edge frugivores and omnivores |
| *Elanoides forficatus* | Non-endemic | > 200 km | 3 - 4 | Low | Low Lands | Carnivores |
| *Cyanocorax chrysops* | Non-endemic | > 200 km | 3 - 4 | Medium | Low Lands | Large Canopy Frug. and Omn. |
| *Colaptes melanochloros* | Non-endemic | > 200 km | 3 - 4 | Medium | Low Lands | Large Canopy insectivores |
| *Ictinia plumbea* | Non-endemic | > 200 km | 3 - 4 | High | Low Lands | Large Canopy insectivores |
| *Myiarchus ferox* | Non-endemic | > 200 km | 3 - 4 | Medium | Low Lands | Edge insectivores |
| *Cyclarhis gujanensis* | Non-endemic | > 200 km | 3 - 4 | High | Low Lands | Canopy insectivores |
| *Myiopagis viridicata* | Non-endemic | > 200 km | 3 - 4 | Medium | Low Lands | Canopy insectivores |
| *Icterus cayanensis* | Non-endemic | > 200 km | 3 - 4 | Medium | Low Lands | Edge frugivores and omnivores |

**S1 Appendix – continuation**

| **Species** | **Endemism** | **Limit of Distribution** | **Type of forest** | **Relative abundance** | **Center of abundance** | **Guilds** |
| --- | --- | --- | --- | --- | --- | --- |
| *Thraupis sayaca* | Non-endemic | > 200 km | 3 - 4 | High | Low Lands | Edge frugivores and omnivores |
| *Aramides cajanea* | Non-endemic | > 200 km | 3 - 4 | Medium | Low Lands | Large ground insect. and frug. |
| *Leptotila verreauxi* | Non-endemic | > 200 km | 5 - 6 | High | High Lands | Edge frugivores and omnivores |
| *Patagioenas cayennensis* | Non-endemic | > 200 km | 5 - 6 | High | Low Lands | Large Canopy Frug. and Omn. |
| *Piaya cayana* | Non-endemic | > 200 km | 5 - 6 | High | Low Lands | Large Canopy insectivores |
| *Cnemotriccus bimaculatus* | Non-endemic | > 200 km | 5 - 6 | Medium | Low Lands | Edge insectivores |
| *Coccyzus melacoryphus* | Non-endemic | > 200 km | 5 - 6 | Medium | Low Lands | Edge insectivores |
| *Megarynchus pitangua* | Non-endemic | > 200 km | 5 - 6 | Medium | Low Lands | Edge frugivores and omnivores |
| *Myiornis auricularis* | Endemic | < 200 km | 1 - 2 | Medium | Low Lands | Edge insectivores |
| *Melanerpes flavifrons* | Endemic | < 200 km | 1 - 2 | Medium | Low Lands | Canopy insectivores |
| *Conopophaga lineata* | Endemic | > 200 km | 3 - 4 | High | High Lands | Understory insectivores |
| *Basileuterus flaveolus* | Non-endemic | < 200 km | 1 - 2 | Medium | High Lands | Understory insectivores |
| *Ara chloropterus* | Non-endemic | < 200 km | 1 - 2 | Medium | Low Lands | Large Canopy Frug. and Omn. |
| *Hemitriccus margaritaceiventer* | Non-endemic | < 200 km | 1 - 2 | High | Low Lands | Edge insectivores |
| *Casiornis rufus* | Non-endemic | < 200 km | 1 - 2 | Medium | Low Lands | Understory insectivores |
| *Dromococcyx pavoninus* | Non-endemic | < 200 km | 1 - 2 | Low | Low Lands | Understory insectivores |
| *Todirostrum plumbeiceps* | Non-endemic | < 200 km | 3 - 4 | Medium | High Lands | Edge insectivores |
| *Galbula ruficauda* | Non-endemic | < 200 km | 3 - 4 | High | Low Lands | Understory insectivores |
| *Picumnus albosquamatus* | Non-endemic | < 200 km | 3 - 4 | Medium | Low Lands | Canopy insectivores |
| *Thamnophilus pelzelni* | Non-endemic | < 200 km | 5 - 6 | High | Low Lands | Edge insectivores |
| *Momotus momota* | Non-endemic | < 200 km | 5 - 6 | High | Low Lands | Understory insectivores |
| *Brotogeris chiriri* | Non-endemic | > 200 km | 1 - 2 | Medium | High Lands | Edge frugivores and omnivores |

**S1 Appendix – continuation**

| **Species** | **Endemism** | **Limit of Distribution** | **Type of forest** | **Relative abundance** | **Center of abundance** | **Guilds** |
| --- | --- | --- | --- | --- | --- | --- |
| *Penelope superciliaris* | Non-endemic | > 200 km | 1 - 2 | Medium | Low Lands | Large Canopy Frug. and Omn. |
| *Ramphastos toco* | Non-endemic | > 200 km | 1 - 2 | Medium | Low Lands | Large Canopy Frug. and Omn. |
| *Tityra cayana* | Non-endemic | > 200 km | 1 - 2 | Medium | Low Lands | Large Canopy Frug. and Omn. |
| *Tityra inquisitor* | Non-endemic | > 200 km | 1 - 2 | Medium | Low Lands | Large Canopy Frug. and Omn. |
| *Thamnophilus doliatus* | Non-endemic | > 200 km | 1 - 2 | High | Low Lands | Edge insectivores |
| *Thamnophilus caerulescens* | Non-endemic | > 200 km | 3 - 4 | High | High Lands | Understory insectivores |
| *Colonia colonus* | Non-endemic | > 200 km | 3 - 4 | Medium | High Lands | Canopy insectivores |
| *Micrastur semitorquatus* | Non-endemic | > 200 km | 3 - 4 | Low | Low Lands | Carnivores |
| *Aratinga leucophthalma* | Non-endemic | > 200 km | 3 - 4 | High | Low Lands | Large Canopy Frug. and Omn. |
| *Pteroglossus castanotis* | Non-endemic | > 200 km | 3 - 4 | High | Low Lands | Large Canopy Frug. and Omn. |
| *Melanerpes candidus* | Non-endemic | > 200 km | 3 - 4 | Low | Low Lands | Large Canopy insectivores |
| *Arremon flavirostris* | Non-endemic | > 200 km | 3 - 4 | Medium | Low Lands | Edge insectivores |
| *Crotophaga major* | Non-endemic | > 200 km | 3 - 4 | Medium | Low Lands | Edge insectivores |
| *Legatus leucophaius* | Non-endemic | > 200 km | 3 - 4 | Medium | Low Lands | Edge insectivores |
| *Lathrotriccus euleuri* | Non-endemic | > 200 km | 3 - 4 | Medium | Low Lands | Understory insectivores |
| *Conirostrum speciosum* | Non-endemic | > 200 km | 3 - 4 | High | Low Lands | Canopy insectivores |
| *Myiarchus swainsoni* | Non-endemic | > 200 km | 3 - 4 | Medium | Low Lands | Canopy insectivores |
| *Pachyramphus polychopterus* | Non-endemic | > 200 km | 3 - 4 | High | Low Lands | Canopy insectivores |
| *Veniliornis passerinus* | Non-endemic | > 200 km | 3 - 4 | High | Low Lands | Canopy insectivores |
| *Camptostoma obsoletum* | Non-endemic | > 200 km | 3 - 4 | Medium | Low Lands | Edge frugivores and omnivores |
| *Coryphospingus cucullatus* | Non-endemic | > 200 km | 3 - 4 | High | Low Lands | Edge frugivores and omnivores |

**S1 Appendix – continuation**

| **Species** | **Endemism** | **Limit of Distribution** | **Type of forest** | **Relative abundance** | **Center of abundance** | **Guilds** |
| --- | --- | --- | --- | --- | --- | --- |
| *Euphonia chlorotica* | Non-endemic | > 200 km | 3 - 4 | High | Low Lands | Edge frugivores and omnivores |
| *Hemithraupis guira* | Non-endemic | > 200 km | 3 - 4 | High | Low Lands | Edge frugivores and omnivores |
| *Myiodynastes maculatus* | Non-endemic | > 200 km | 3 - 4 | High | Low Lands | Edge frugivores and omnivores |
| *Myiozetetes similis* | Non-endemic | > 200 km | 3 - 4 | High | Low Lands | Edge frugivores and omnivores |
| *Saltator similis* | Non-endemic | > 200 km | 3 - 4 | High | Low Lands | Edge frugivores and omnivores |
| *Turdus amaurochalinus* | Non-endemic | > 200 km | 3 - 4 | High | Low Lands | Edge frugivores and omnivores |
| *Turdus leucomelas* | Non-endemic | > 200 km | 3 - 4 | High | Low Lands | Edge frugivores and omnivores |
| *Chlorostilbon lucidus* | Non-endemic | > 200 km | 3 - 4 | High | Low Lands | NA |
| *Glaucidium brasilianum* | Non-endemic | > 200 km | 3 - 4 | High | Low Lands | Carnivores |
| *Herpetotheres cachinnans* | Non-endemic | > 200 km | 5 - 6 | Medium | Low Lands | Carnivores |
| *Rupornis magnirostris* | Non-endemic | > 200 km | 5 - 6 | High | Low Lands | Carnivores |
| *Dryocopus lineatus* | Non-endemic | > 200 km | 5 - 6 | High | Low Lands | Large Canopy insectivores |

Species not forest-dependent, observed occasionally, not regarded in the analysis:

*Crypturellus parvirostris, Rhynchotus rufescens, Nothura maculosa, Syrigma sibilatrix, Cathartes aura, Coragyps atratus, Elanus leucurus, Polyborus plancus, Milvago chimachima, Aramus guarauna, Cariama cristata, Vanellus chilensis, Columbina talpacoti, Columbina squammata, Aratinga aurea, Crotophaga ani, Guira guira, Tapera naevia, Colaptes campestris, Furnarius rufus, Euscarthmus meloryphus, Donacobius atricapillus, Troglodytes aedon, Ammodramus humeralis, Emberizoides herbicola* and *Sporophila angolensis.*

Forest species which did not fit properly to the sampling methodology, not regarded in the analysis:

*Caprimulgus rufus,Caprimulgus parvulus, Nyctidromus albicollis, Nyctiphrynus ocellatus, Sarcoramphus papa, Synallaxis ruficapilla, Gnorimopsar chopi, Nyctibius griseus ,Coereba flaveola, Pipra fasciicauda, Otus choliba, Phaethornis eurynome* and *Thalurania glaucopis*.

NA – Not analyze
